# Analysis of college students' attitudes toward the use of ChatGPT in their academic activities: effect of intent to use, verification of information and responsible use

**DOI:** 10.1186/s40359-024-01764-z

**Published:** 2024-05-08

**Authors:** Benicio Gonzalo Acosta-Enriquez, Marco Agustín Arbulú Ballesteros, Olger Huamaní Jordan, Carlos López Roca, Karina Saavedra Tirado

**Affiliations:** 1 Universidad Nacional de Trujillo, Trujillo, Perú; 2 Universidad Tecnológica del Perú, Lima, Perú; 3 Universidad Nacional Mayor de San Marcos, Lima, Perú

**Keywords:** Artificial intelligence, ChatGPT, Attitude, Acceptance, Intention, Emotions, College students, Education, Higher, Curriculum, Educational technology, UTAUT2, Behavior, Responsible use, Frequency of use, Information verification, Risk, Boredom

## Abstract

**Background:**

In recent years, the use of artificial intelligence (AI) in education has increased worldwide. The launch of the ChatGPT-3 posed great challenges for higher education, given its popularity among university students. The present study aimed to analyze the attitudes of university students toward the use of ChatGPTs in their academic activities.

**Method:**

This study was oriented toward a quantitative approach and had a nonexperimental design. An online survey was administered to the 499 participants.

**Results:**

The findings of this study revealed a significant association between various factors and attitudes toward the use of the ChatGPT. The higher beta coefficients for responsible use (β=0.806***), the intention to use frequently (β=0.509***), and acceptance (β=0.441***) suggested that these are the strongest predictors of a positive attitude toward ChatGPT. The presence of positive emotions (β=0.418***) also plays a significant role. Conversely, risk (β=-0.104**) and boredom (β=-0.145**) demonstrate a negative yet less decisive influence. These results provide an enhanced understanding of how students perceive and utilize ChatGPTs, supporting a unified theory of user behavior in educational technology contexts.

**Conclusion:**

Ease of use, intention to use frequently, acceptance, and intention to verify information influenced the behavioral intention to use ChatGPT responsibly. On the one hand, this study provides suggestions for HEIs to improve their educational curricula to take advantage of the potential benefits of AI and contribute to AI literacy.

**Supplementary Information:**

The online version contains supplementary material available at 10.1186/s40359-024-01764-z.

## Introduction

Artificial intelligence (AI) applications for education are being developed at an increasing rate [1]. Moreover, its application in education has increased considerably worldwide in the digital era [2]. In this sense, the adoption of artificial intelligence educational technologies in higher education institutions (HEIs) is expected to increase exponentially, transforming teaching and learning [1, 3].

In late 2022, OpenAI released the third version of the generative pretrained transformer, ChatGPT, which is a language model capable of understanding human input and producing response text nearly indistinguishable from natural human language [4]. It is also designed to generate human-like text in a conversational style, highlighting its ability to perform a wide range of linguistic tasks, such as translation, summarization, response, and text generation [5]. It is also pretrained with a large text dataset (including books, articles, and websites) through a language modeling task [6], through which it learns patterns and relationships between words and phrases in natural language to generate coherent and realistic responses in a conversation [4].

The immersion of AI in education has several implications, such as achieving goal 4 related to quality education [7], enabling new approaches to assessment and teaching, and allowing access to chatbots and virtual assistants capable of delivering more personalized learning [8, 9]. Chatbots can play multiple advisory roles in HEIs [10, 11] and help students improve their understanding of text by posing personalized queries, providing answers, and serving as a source of information for many aspects of university life, including module schedules and organization [1, 12, 13].

The number of ChatGPT users has increased exponentially, spreading the popularity of this tool in a variety of settings, especially in education [14]. Educators and universities are taking steps to mitigate their usage in academic settings [15].

Since its launch on November 30, 2022, ChatGPT has become the fastest growing user application in history, reaching 100 million active users by early 2023 [16]. Moreover, in Japan, 55.6% of respondents were more or less inclined to use this chatbot in the future [17].

By early 2023, 12% of respondents reported having used ChatGPT to generate text themselves, and 38% reported having seen text generated by artificial intelligence technology for someone else [18]. Globally, the ChatGPT is widely accepted among 25- to 34-year-olds. However, those under the age of 24 are the second largest user base, and together with those under the age of 34, they account for more than 60% of ChatGPT users because younger age groups tend to explore new technologies more than older age groups [19].

In recent years, the use of ChatGPT in educational settings has gained significant attention from researchers. Recent studies have explored students' attitudes towards this artificial intelligence tool and the factors influencing its adoption. [20] found that students at the University of Jordan exhibited a positive attitude towards using ChatGPT as a learning tool, although they expressed concerns about the accuracy of the information provided. [21] identified habitual use, performance expectancy, and hedonic motivation as significant predictors of students' intentions to use ChatGPT in Poland. [22] highlighted the importance of trust in adopting new technologies, demonstrating that perceived enjoyment and informativeness significantly influenced students' attitudes towards ChatGPT in Bangladesh. [23] found that perceived utility had both direct and indirect impacts on students' intentions to continuously use ChatGPT in South Korea. Additionally, [24] emphasized the need for universities to develop strategies to ensure ethical and responsible use of ChatGPT, addressing the potential for academic dishonesty.

To develop the study, the short version of the Pupils' Attitudes Toward Technology model (PATT-SQ-SE) was employed because it provides items related mainly to measuring the affective component of attitudes toward technology. This approach was deemed appropriate because it classifies emotions into positive and negative emotions and additionally provides items to measure interest, boredom, and importance. Similarly, items from the Mitcham score questionnaire were adapted to evaluate aspects of the cognitive, affective, and behavioral components, logically considering Mitcham's four dimensions of technology, namely, knowledge, volition, activity, and object [25, 26]. Therefore, the use of the attitude components from Mitcham's philosophical framework of technology is justified, as it contributes constructs classified into affective, cognitive, and behavioral components, providing a reference framework for measuring students' attitudes toward a specific technology [27].

Furthermore, the use of the UTAUT2 model was considered pertinent for two reasons. First, it integrates constructs widely used by various academics to assess the acceptance and attitudes of users toward a technology, such as perceived risk, ease of use, and intention to use it frequently [28]. Second, this model is used to evaluate the adoption and initial use of a technology in introductory phases [29], as is the case with generative AI such as ChatGPT. The primary objective of this study is to analyze the attitudes of university students toward ChatGPT. The significance of this study is twofold. First, we empirically strengthen the UTAUT2 and PATT-SQ-SE models by incorporating new theoretical constructs for evaluating attitudes toward generative AI tools (such as the ChatGPT) in the Peruvian context. Second, we analyze the effects of the intention to use frequently and of information verification on responsible use by students.

The specific objectives of the current study are as follows:

1. Determine how the cognitive component constructs are predictors of the behavioral component.
2. Determine how the constructs of the affective component predict the behavioral component.
3. Determine how the intended use and verification of information influence the responsible use of the ChatGPT.

Despite the growing popularity of ChatGPT in educational settings and its potential to transform how students learn and interact with technology [1, 2], there is a notable lack of empirical studies specifically addressing the measurement of college students' attitudes toward this tool. It is critical to investigate the factors influencing the intention to use, information verification, and responsible use of ChatGPT by students [11, 30, 31], as these aspects are fundamental for successful and ethical implementation of the tool in academic activities [32]. This study aims to fill this knowledge gap by providing a solid foundation for future research on the impact of ChatGPT in higher education. By exploring college students' attitudes toward ChatGPT, this study contributes to understanding how higher education institutions can effectively integrate this technology into teaching and learning processes [3], address ethical issues, and develop students' digital literacy skills [8, 9]. The results of this study will be relevant to educators, administrators, and policymakers seeking to leverage the benefits of ChatGPT in higher education [10, 13] while mitigating associated risks and promoting the responsible use of this technology.

Therefore, this study is justified because the use of generative artificial intelligence tools such as ChatGPT is becoming increasingly popular across various sectors and industries, particularly in education. The rapid adoption of this technology by college students highlights the need to understand their attitudes toward ChatGPT, as this will influence how it is integrated and utilized in academic activities. Analyzing students' attitudes is crucial for effectively integrating ChatGPT in various educational contexts and addressing any concerns or issues that may arise. Moreover, incorporating constructs such as the intention to verify information and responsible use of the ChatGPT underscores the importance of promoting ethical and responsible use of these tools, ensuring that they are efficiently utilized in academic activities. Consequently, this study contributes to the ongoing debate about the use of artificial intelligence tools and their impact on higher education, providing valuable insights into college students' attitudes toward ChatGPT.

## Literature review

### Review of attitudes toward technology

An attitude is an evaluation of a psychological object and is represented by dimensions such as good versus bad and pleasant versus unpleasant [27, 33]. Furthermore, it is the mental disposition of a person to develop certain behaviors [34]. Traditionally, attitude has been divided into affective, cognitive and behavioral components [35–37].

Attitudes toward technology are based on a person's beliefs, and these beliefs influence his or her behavior [38]. Consequently, the formation of attitudes toward technology depends on an individual's underlying ideas, which then influence their behavioral patterns.

This study presupposes that attitude combines cognitive (beliefs, experiences, and opinions regarding ChatGPT), affective (emotions toward ChatGPT), and behavioral (behavioral predispositions toward ChatGPT use) elements [39].

### Review of UTAUT2 theory and the PATT-SQ-SE model for measuring attitudes toward generative AI

In the context of technology adoption models, the unified theory of acceptance and use of technology (UTAUT2) stands as a robust and adaptable reference framework, justifying its use for assessing university students' attitudes toward generative AI.

The UTAUT2 model, an extension of the original model, incorporates additional constructs such as hedonic motivation, price value, habit, and usage intention, along with the basic elements of performance expectancy, effort expectancy, social influence, and facilitating conditions, thereby expanding its predictive power. Researchers have consistently utilized UTAUT2 in various contexts to measure user acceptance and attitudes toward specific technologies [28, 40–42].

[43] applied UTAUT2 to understand higher education students' attitudes toward AI and found that performance expectancy and facilitating conditions significantly influenced students' behavioral intentions, although effort expectancy was not a major influencer in the context of AI.

A strength of UTAUT2 lies in its adaptability to different technological and research contexts, such as the Peruvian context. In the field of AI, studies have demonstrated how UTAUT2 can be adapted to understanding specific aspects of AI adoption, as in the work of [44], which focused on medical students' perceptions of AI, revealing a positive attitude despite limited training. Thus, these findings suggest that UTAUT2 effectively captures attitudes in specialized fields where AI is rapidly becoming integral, such as healthcare.

Moreover, UTAUT2 has been employed to assess teachers' attitudes toward AI in education; however, while teachers are positively inclined toward AI, its integration into teaching practices is limited. This highlights UTAUT2's capacity to provide insights into the barriers to and facilitators of AI adoption among different user groups within higher education [45].

The specific application of UTAUT2 to AI in higher education highlights the critical factors influencing AI adoption. [46] found that higher education students from certain disciplines are eager to learn more about AI, signaling an unmet need for AI education and training. Additionally, [42] identified specific gender differences in AI adoption among preservice teachers, with the perception of ease of use and utility being crucial. These findings signal the importance of customizing AI education initiatives to cater to the diverse needs and perceptions of higher education students.

In this specific study, items from the constructs of ease of use, perceived risk, and intention to use frequently were adapted to assess attitudes toward ChatGPT among university students for their versatility and ease of prediction, as demonstrated in the study by [28].

On the other hand, the study of attitudes toward technology (PATT) has a long history and was developed in the 1980s by Roat and de Vries [47] to explore students' interest and attitudes toward technology. Over the years, the PATT questionnaire has undergone numerous validation and reliability tests and has been used with students from different countries in Africa, Asia, and new European countries who are undergoing various modifications and improvements [48]. [49] modified the survey, resulting in a shortened questionnaire with fewer items called the PATT-SQ. However, this version brought linguistic adaptation issues in other contexts and reported difficulties as students tended to use the midpoint option of the items (affecting average scores). Consequently, [27] contributed to the PATT-SQ-SE instrument, which, in addition to including items from previous versions, emphasizes constructs such as interest and boredom. A person's knowledge can become an object of interest at any moment; thus, interest is directed toward something [50], such as using a technology, and can become a motivating factor for engaging with it [51]. Boredom, on the other hand, refers to an emotional state where the individual perceives a lack of interest or low level of stimulation in the activity in question. This mood can lead to reduced engagement and a decrease in motivation to continue with technological activity [27].

### OpenAI and the development of ChatGPT

OpenAI is an organization that has developed generative AI projects such as ChatGPT [52], which includes four versions (GPT1, GPT2, GPT3, and GPT4). However, after its most successful version (GPT-3), OpenAI has continued its research and development, launching GPT-4 [4, 53].

As a language model, ChatGPT is not recent, as GPT-1 was released in 2018 based on the transformer architecture or neural network architecture designed for natural language processing tasks, such as language modeling and machine translation. It was also previously trained on a text dataset (including books, articles, and webpages) using a language modeling task [54].

Its successor, GPT-2, was also trained with a massive amount of text data heap using a language-modeling task. However, unlike in the previous version, longer text sequences consistent with human language could be generated [55]. 

The GPT-3 version, which has 175 billion parameters and was trained on a massive corpus of text data (including webpages, books, and other written materials) using a language-modeling task [56], can generate text of high quality, coherence, and realism in natural language. Its main virtue is its ability to perform a wide range of natural language processing tasks, such as text classification, opinion analysis, and complex question answering [4, 57]. The GPT-3 has a free version; therefore, it is the most popular among college students.

The GPT-4 version is a multimodal language model with the ability to accept image-text inputs and generate text outputs, demonstrating human-level performance on several academic and professional benchmarks [58, 59].

### Conceptual model and research hypothesis

**Fig. 1:**
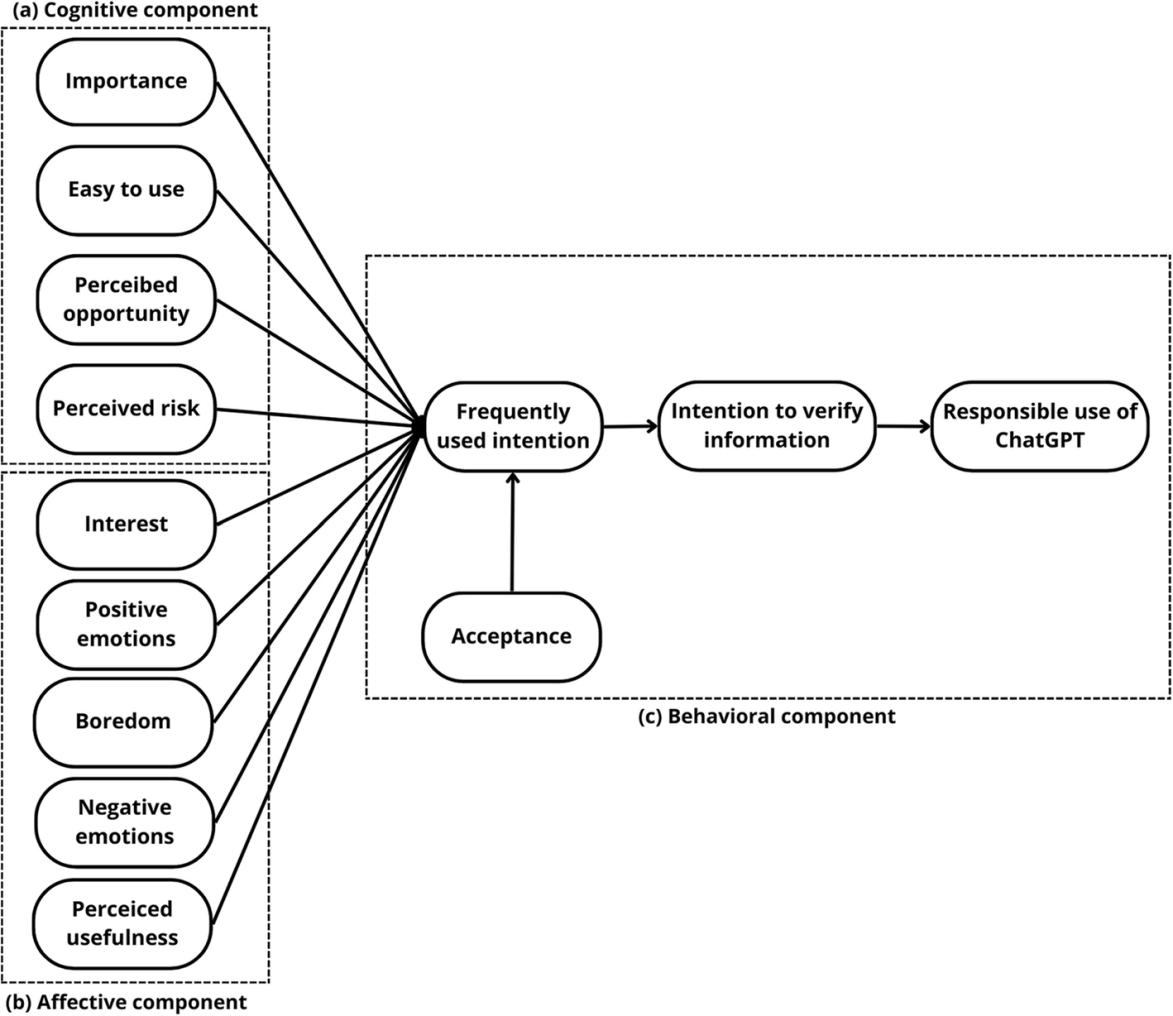
Research model, where the solid lines indicate the direct effect and the dotted lines represent the three attitude components

Figure 1 illustrates the research model based on relationships established by hypotheses of previous studies proposing the unified theory of acceptance toward ChatGPT-UTAC, where 13 constructs are incorporated based on the attitude components of Mitcham's philosophical framework of technology [60] and the PATT-SQ-SE model [48], where the directions of the relationships are oriented as follows: the cognitive component affects the affective component, which in turn influences the behavioral component. In addition, the first two variables determine subjects’ behavioral intentions [27, 61].

In summary, this study develops a hybrid model aimed at exploring and testing new cause‒effect relationships among constructs provided by the unified theory of acceptance (UTAUT2), the attitudinal components of Mitcham's philosophical framework of technology, and the PATT-SQ-SE model. The goal is to gain a deeper understanding of how cognitive and affective components influence and predict students' behavior when using the ChatGPT.

The constructs of significance, opportunity, ease of use, and perceived risk, as well as their relationships with the intention to use, combined the PATT-SQ-SE model with the UTAUT2 theory, which provides the construct of frequent use intention. From the perspective of Mitcham's philosophical framework of technology, these are situated within the cognitive component of the attitude toward the ChatGPT. Moreover, these relationships enable the evaluation of how cognitive component constructs are predictors of the behavioral component of students' attitudes toward ChatGPT.

On the other hand, constructs of interest, boredom, perceived utility, and positive and negative emotions—following the guidance of Mitcham's philosophical framework of technology—were placed in the affective component of the attitude. This combines the PATT-SQ-SE model with the intention of use from the UTAUT2 theory, facilitating the determination of how affective component constructs predict the behavioral component. Additionally, the relationships between UTAUT2 theory and the intention to verify information and the responsible use of this technology were included; these relationships are located within Mitcham's philosophical framework of technology in the behavioral component.

### Hypotheses from the constructs of the cognitive component of attitude toward technology

Perceived importance (PIM) refers to students' general beliefs about technology and is linked to the cognitive component [27, 48]. The perceived importance of a technological tool affects the intention to use it in the future [62].

Ease of use (EUS) has been employed in studies of technology adoption, and its significant influence on the user's intention to use a technology has been determined [40, 63]. [64], using structural equation modeling, corroborated the relationship between ease of use and perceived usefulness (PUS) and between ease of use and the intention to use technological tools. Furthermore, in India [30, 31, 65], ease of use and perceived usefulness were employed, confirming the positive association between these variables and user behavioral intention.

Previous studies have suggested that perceived opportunity (PO) significantly influences students’ intention to frequently use innovative technologies [1]. According to [63], security risk has a negative impact on users’ intention to use. Likewise, perceived risk is significantly associated with the intention to use and reduces the user's willingness to adopt new technology tools, such as artificial intelligence [66, 67]. Additionally, there is a potential concern that students are likely to be aware of the frequent use of substantial amounts of personal information. Therefore, the preservation of data privacy and security is important [11, 68, 69]. Security concerns may affect the use of chatbots [70].

- Hypothesis 1. Perceived importance-PIM positively influences users’ intention to frequently use ChatGPT.
- Hypothesis 2. The ease of use of EUS positively influences user intention to use ChatGPT frequently.
- Hypothesis 3. Perceived opportunity-POP positively influences users’ intention to use ChatGPT frequently.
- Hypothesis 4. Perceived risk-PRI positively influences users’ intention to use ChatGPT frequently.
- Hypothesis 7. Perceived usefulness-PUS positively influences users’ intention to frequently use ChatGPT.

### Hypotheses from the constructs of the affective component of attitude toward technology

Interest can change and develop over time as new knowledge is acquired, allowing for a shift from situational to individual interest [27, 71]. According to [60], interest influences the intention to frequently use a technological tool.

Boredom (BORE) can affect students’ intentions to use a technology; therefore, software design companies have been developing intuitive and easy-to-use tools to prevent users from experiencing a sense of boredom [27].

[72] designed a scale of attitudes toward AI in which they formulated positive items related to the opportunities and benefits of AI to measure positive emotions; they also formulated negative items related to the main concerns about AI to measure negative emotions. Participants endorsed some positive statements with high frequency; for example, that there would be many beneficial applications of AI but were less willing to state whether AI was better than humans in complex decisions. On negative items, many felt that AI could threaten job security, but a few instinctively disliked AI or found it sinister.

- Hypothesis 5. Interests-INTERESTS positively influences users’ intention to frequently use ChatGPT.
- Hypothesis 6. Boredom-BORE negatively influences users’ intention to use ChatGPT frequently.
- Hypothesis 8. Positive emotions: POSEMO positively influences users’ intention to use ChatGPT frequently.
- Hypothesis 9. Negative emotions: NEGEMO negatively influences users’ intention to use ChatGPT frequently.

### Hypotheses from the constructs of the behavioral component of attitude toward technology

Technology acceptance focuses primarily on the willingness to adopt technology through consumer choice [64]. In addition, the acceptance of an information system generally refers to users’ decisions on whether to purchase or implement the system in the future, in the sense of active willingness and not only in the sense of reactive tolerance [73, 74]. In this study, we explored the moderating effect of acceptance on intention to use, with the understanding that a moderating variable affects the relationship between independent and dependent variables [28].

Intention to use is the main construct of UTAUT2, representing the degree of willingness and effort of individuals to perform the underlying behavior [75, 76]. [77] conducted a meta-analysis on the UTAUT and reported a significant relationship between frequent use intention and technology tool use. In addition, GPT-3 is not completely free of bias in the answers it provides and may provide incorrect information with fabricated data [32]; therefore, it is advisable to verify the information obtained.

Based on the relationships proven by [39, 78–81], it is hypothesized that the intention to verify information influences the responsible use of the ChatGPT. [82] provided guidelines for the use of the ChatGPT in education, prioritizing its responsible and ethical use.

- Hypothesis 10. Acceptance-ACCEP positively influences user intention to frequently use the ChatGPT.
- Hypothesis 11. The intention to frequently use INTU positively influences users’ intention to verify the information obtained in ChatGPT.
- Hypothesis 12. The intention to verify information, INVERINFO, positively influences responsible use, RESPONUSE ChatGPT.

## Method

The study was oriented toward a quantitative approach and had a nonexperimental design of the applied type. Thus, an empirical study was conducted to test the research hypotheses presented in the literature review process on students' attitudes toward a GAN system. For this purpose, a questionnaire [28] was designed and administered to university students who expressly indicated that they had implemented ChatGPT in their academic activities.

### Participants

A study involving 499 university students from 10 public and private universities in Peru was conducted through no probabilistic convenience sampling, during which all participants voluntarily agreed to participate [83]. This sampling method was justified considering the limitations of time and resources. Hence, by employing no probabilistic convenience sampling, access was gained to a diverse and considerably large sample of university students from various academic fields, ensuring the reliability of the results. Although this sampling method does not guarantee the statistical representativeness of the total university population, it provides valuable and directional insights into attitudes toward ChatGPT among students.

Regarding sociodemographic characteristics (as shown in Table 1), a notable diversity and inclusive spirit among the student community is evident, with a slightly greater proportion of women (52%) than men (48%) participating in the study. Moreover, young people between 17 and 22 years old, who constitute the unit of analysis for this study, show a significant inclination toward new technologies, such as ChatGPT, demonstrating its role in advancing toward a digital era. Additionally, the participants’ ages ranged from 19 to 22 years, suggesting that they were working midway through their academic training.

**Table 1:** Sociodemographic profile of the sample (*n*=499)

**Item**		**N**	**%**
Gender	Male	238	48
	Female	261	52
Age	17-18	74	20
	19-20	111	30
	21-22	185	50
	23-25	67	13.4
	26-35	43	8.6
	36-45	10	2
	46 years and older	9	1.8
Type of university	Public	291	58
	Private	208	42
University Career	Administration	20	4
	Architecture and urbanism	3	0.60
	Political science	2	0.40
	Biological sciences	3	0.60
	Communication Sciences	3	0.60
	Accounting and finance	3	0.60
	Law	13	2.60
	Early childhood education	22	4.5
	Primary education	29	5.81
	Secondary education	126	25.2
	Health sciences	53	10.6
	Engineering	77	15.5
	Social sciences	4	0.80
	Other	141	28.2
Do you use ChatGPT in your academic activities?	Yes	499	100
	No	0	0

The analysis also highlights an interesting distribution between students from public universities (58%) and private universities (42%), with a predominance of the former. This provides relevant information regarding the widespread adoption and acceptance of the ChatGPT in higher education in Peru. Furthermore, Table 1 reveals that the participants come from a variety of academic disciplines, from business administration to engineering and health sciences, which is significant because it allows us to evaluate attitudes toward the ChatGPT from the perspective of students in various fields.

Finally, it is pertinent to mention that all the study participants had prior experience using the ChatGPT. Its widespread use indicates a move toward more interactive and personalized learning methods, preparing students for a future where artificial intelligence will play a key role in contemporary education.

All ethical procedures were approved by all universities selected for the study, and all participants provided informed consent.

### Instruments

Based on the literature review and identification of the theoretical constructs, 45 items were formulated with a Likert scale of 5 response options, ranging from "strongly disagree" to "strongly agree"; this type of scale was used because of its relevance and adaptability to measure attitude [84]. Additionally, the choice of a 5-point Likert scale over a 7-point scale is justified primarily by its simplicity and ease of comprehension and by the reduction in respondent fatigue. A shorter scale is generally easier to understand, allowing respondents to make quick and clear decisions. This, in turn, reduces fatigue by offering fewer choices, which is crucial for maintaining engagement and accuracy in responses, especially in lengthy or complex surveys. These factors contributed to the overall effectiveness of the data collection and the quality of the responses obtained.

Thirteen constructs were assessed (Appendix 1). The importance, perceived interest and boredom scales were adapted from [27]. The ease of use, perceived risk and frequently used intention constructs were adapted from [28] of the UTAUT2 model. The scores for opportunity and positive and negative emotions were adapted from the general attitudes toward artificial intelligence scale (GAAIS) of [1]. Importance, interest and boredom were adapted from the short version of the PATT-SQ-SE questionnaire [48]. Acceptance and intention to verify information were adapted from the general attitudes toward AI scale of [85]. Finally, the construct responsible for use was adapted from the IT use scale of [86], and the guidelines for ChatGPT use in higher education [82] were used as a reference.

The survey was consolidated in an online form for its application, where in addition to the items of the constructs, sociodemographic questions such as age, gender, type of university, current university major, and a filter question referring to the implementation of the ChatGPT in the academic activities of the respondents were attached.

Before administering the survey, the instrument was evaluated by six experts in the field to determine the relevance, clarity, representativeness, coherence, and consistency of the wording of all the items. In addition, a pilot test with 30 students was conducted to assess the validity and reliability of the scale. The final application of the survey was subsequently conducted with the final wording of the items.

### Data collection and analysis method

Data were collected by administering an online survey to undergraduate students from ten public and private universities in Peru in August 2023. The average time to complete the form was 15 min. A total of 507 responses were collected from the participants; however, 499 responses were used, rejecting eight forms because the participants did not agree to participate by selecting the option "no, I don’t accept.” In a mandatory branching question, they indicated that they had not used the ChatGPT in their academic activities.

Structural equation modeling was performed with SmartPLS-v4 [87], which is based on the partial least squares (PLS) technique, to test the theoretical model. Reliability was assessed using Cronbach's alpha coefficient [88], while convergent and discriminant validity were assessed with factor loadings and composite reliability, whose values were above 0.7, and with the estimate of the average variance extracted (AVE), where most of the values were greater than 0.5. The model demonstrated reliability and convergent validity based on the measures presented (Table 4). Likewise, to evaluate discriminant validity, the root of the AVE of each construct was evaluated by analyzing whether its values were greater than the correlations of all the other constructs and the specific construct [88]. Table 5 presents the convergent and discriminant validity reports, which indicate good conditions for the measurement instrument.

## Results

### Quality testing of the measuring instrument

The main quality tests used to determine the validity and reliability of the measurement instrument are as follows.

### Standardized list

To analyze the measurement instrument, the relationships between a series of items and their respective standardized external loadings were examined. These external loadings represent the contributions of each item to an underlying latent construct. In the Supplementary Material Table, it is observed that several items have high and significant external loadings on specific constructs, suggesting that these items are strongly associated with those constructs. The standardized external loadings indicate the strength and direction of the relationship between the items and the constructs. A high external loading (near 1) indicates a strong, positive relationship, whereas a low external loading (near 0) suggests a weak or no relationship.

### Relationships between different constructs of the study

In Table 2, the values in the matrix represent the correlations between the pairs of constructs assessed. A correlation closer to 1 indicates a strong positive relationship, while a correlation close to -1 indicates a strong negative relationship. A correlation close to 0 suggested a weak or null relationship.

**Table 2:** Correlations between constructs

**Constructs**	**BORE**	**ACCEP**	**NEGEMO**	**POSEMO**	**EUS**	**PIM**	**INTU**	**INVERINFO**	**INTERES**	**POP**	**PRI**	**RESPONUSE**	**PUS**
**BORE**	1.000	0.000	0.000	0.000	0.000	0.000	-0.145	-0.074	0.000	0.000	0.000	-0.059	0.000
**ACCEP**	0.000	1.000	0.000	0.000	0.000	0.000	0.441	0.225	0.000	0.000	0.000	0.181	0.000
**NEGEMO**	0.000	0.000	1.000	0.000	0.000	0.000	-0.017	-0.008	0.000	0.000	0.000	-0.007	0.000
**POSEMO**	0.000	0.000	0.000	1.000	0.000	0.000	0.418	0.213	0.000	0.000	0.000	0.172	0.000
**EUS**	0.000	0.000	0.000	0.000	1.000	0.000	0.204	0.104	0.000	0.000	0.000	0.084	0.000
**PIM**	0.000	0.000	0.000	0.000	0.000	1.000	0.193	0.098	0.000	0.000	0.000	0.079	0.000
**INTU**	-0.145	0.441	-0.017	0.418	0.204	0.193	1.000	0.509	0.238	-0.002	-0.104	0.411	-0.033
**INVERINFO**	-0.074	0.225	-0.008	0.213	0.104	0.098	0.509	1.000	0.121	-0.001	-0.053	0.806	-0.017
**INTERES**	0.000	0.000	0.000	0.000	0.000	0.000	0.238	0.121	1.000	0.000	0.000	0.098	0.000
**POP**	0.000	0.000	0.000	0.000	0.000	0.000	-0.002	-0.001	0.000	1.000	0.000	-0.001	0.000
**PRI**	0.000	0.000	0.000	0.000	0.000	0.000	-0.104	-0.053	0.000	0.000	1.000	-0.043	0.000
**RESPONUSE**	-0.059	0.181	-0.007	0.172	0.084	0.079	0.411	0.806	0.098	-0.001	-0.043	1.000	-0.014
**PUS**	0.000	0.000	0.000	0.000	0.000	0.000	-0.033	-0.017	0.000	0.000	0.000	-0.014	1.000

### Proportion of variability in the constructs

In this context of analysis, the R-squared ${{\varvec{v}}{\varvec{a}}{\varvec{l}}{\varvec{u}}{\varvec{e}}{\varvec{s}}({\varvec{R}}}^{2})$ indicate the proportion of variability in the constructs that can be explained by the independent variables considered in the model. The higher the value of R^2^ is, the more adequately the independent variables explain the variations in the corresponding construct. Table 3 shows certain values for the INTU construct, the value of which is ${{\varvec{R}}}^{2}=0.538$. This finding suggested that approximately 53.8% of the variability observed in *frequent use intention* can be explained by the independent variables included in the analysis. In other words, these variables have a significant impact on the variability in the intention to frequently use the system or intervention under study.

**Table 3:** Proportion of variability in the constructs

**Constructs**	${{\varvec{R}}}^{2}$	**%**
INTU	0.538	53.8
INVERINFO	0.259	25.9
RESPONUSE	0.650	65.0

For the INVERINFO construct, the value of ${{\varvec{R}}}^{2}$=0.259. This means that approximately 25.9% of the variability in the *intention to verify information* can be explained by the independent variables considered. Although this value is lower than that in the previous case, this still indicates that the independent variables contribute significantly to the variability in the intention to verify information.

For the RESPONSE construct, ${{\varvec{R}}}^{2}=0.650$. This finding implies that approximately 65% of the variability observed in the *use of ChatGPT* can be explained by the independent variables analyzed. These findings indicate that these variables strongly influence the variability in the degree of responsible use of the ChatGPT system.

In summary, the values of ${{\varvec{R}}}^{2}$ provide valuable information on the ability of the independent variables to explain the variability in the constructs of interest. These values indicate the extent to which the variables included in the analysis contribute to the understanding and prediction of the behaviors and attitudes associated with each construct.

### Reliability and validity of the construct

In the present analysis, the psychometric properties of the constructs under consideration were evaluated. The reliability and internal consistency of the items that compose each construct were evaluated by means of several metrics. The results revealed the following:

In Table 4, the constructs present a high level of internal consistency, measured by Cronbach's alpha coefficient. The standardized and unstandardized values of Cronbach's alpha remained consistent for each construct, ranging from 0.612 to 0.977.

**Table 4:** Reposits of the validity and reliability of the construct

**Constructs**	α	**CR**	**AVE**
BORE	0.885	0.887	0.723
ACCEP	0.977	0.977	0.913
NEGEMO	0.862	0.864	0.678
POSEMO	0.931	0.931	0.817
EUS	0.919	0.920	0.742
PIM	0.969	0.969	0.887
INTU	0.965	0.913	0.777
INVERINFO	0.612	0.712	0.601
INTERES	0.949	0.950	0.826
POP	0.947	0.947	0.857
PRI	0.728	0.726	0.474
RESPONUSE	0.975	0.969	0.864
PUS	0.926	0.926	0.806

α Cronbach's alpha, *CR* Composite reliability, *AVE* Average variance extracted

Composite reliability (CR) is high for all the constructs, reflecting the consistency and precision of the measurements. The values range from 0.712 to 0.977, indicating high reliability in the measurement of the constructs.

The average variance extracted (AVE) measures the proportion of variance in the items that is being captured by the underlying construct. AVE values range from 0.474 to 0.913, suggesting that a considerable amount of variance is explained by the constructs relative to the items. However, although the average variance extracted (AVE) for a single construct yielded a value of 0.475, which is below the conventional threshold of 0.5, it is crucial to note that the assessment of the model's validity is not based solely on this single criterion. Specifically, the model's convergent and discriminant validity provides substantial evidence supporting the integrity of the implicated constructs, where items associated with each construct had a significant and cohesive relationship, indicating strong convergent validity. Additionally, the composite reliability (CR) criterion was used, where all the constructs had values greater than 0.70. This aspect is important because it ensures that despite the slightly low AVE, the concepts are conceptually and empirically distinct from each other and relevant to the proposed theoretical framework.

These findings indicate that the constructs assessed in this study exhibit strong internal consistency, high reliability, and an adequate ability to capture variance in their component items. Taken together, these results validate the robustness and quality of the measurements made in relation to the constructs studied.

### Convergent and discriminant validity

An analysis of the heterotrait–monotrait relationship index test (HTMT) was performed to assess convergent discrimination and discrimination between the different constructs. This test measures the relationship between items of one construct and items of other constructs to determine whether the measures of one construct are more similar to each other than to other constructs.

Table 5 shows that in the main diagonal, the values are all zero because they represent comparisons between items of the same construct. Values above the main diagonal show comparisons between constructs in terms of similarity of measurements. A value significantly less than 1 indicates that convergent discrimination (similarity within the same construct) is better than discriminant discrimination (similarity between different constructs), which is desirable for good convergent and discriminant validation. These values can indicate whether the constructs are effectively measuring different concepts or whether there is a high correlation between the constructs.

**Table 5:** Convergent and discriminant validity report

Constructs	ACCEP	BORE	EUS	INTU	INVERINFO	INTEREST	NEGEMO	POP	PRI	PUS	RESPONUSE	PIM
BORE	0.764											
EUS	0.818	0.809										
INTU	0.848	0.778	0.830									
INVERINFO	0.874	0.655	0.811	0.789								
INTEREST	0.900	0.782	0.865	0.840	0.876							
NEGEMO	0.236	0.304	0.239	0.248	0.165	0.196						
POP	0.833	0.783	0.874	0.815	0.819	0.896	0.240					
PRI	0.064	0.288	0.114	0.146	0.241	0.097	0.455	0.105				
PUS	0.866	0.843	0.804	0.829	0.815	0.890	0.294	0.869	0.130			
RESPONUSE	0.730	0.555	0.739	0.661	0.850	0.751	0.075	0.742	0.149	0.693		
PIM	0.836	0.827	0.810	0.834	0.775	0.876	0.302	0.803	0.144	0.824	0.693	
POSEMO	0.872	0.747	0.853	0.851	0.900	0.866	0.179	0.872	0.079	0.862	0.766	0.847

Overall, in this analysis, the HTMT test values are within an acceptable range, suggesting that the constructs have construct validity and discriminant validity in general. These results are useful for understanding how the constructs relate to each other in terms of their measurements and for assessing the construct validity of the measures used in the study.

### Tests of Collinearity and PLSpredict

Table 6 presents the results of the collinearity analysis using the variance inflation factor (VIF), which indicates moderate multicollinearity in most of the variables analyzed in relation to INTU, as the VIF values remain less than 5. This suggests that the influence of these variables on the intention to use frequently is significant but not problematic from the perspective of collinearity. The relationships INTU→INVERINFO and INVERINFO→RESPONUSE exhibit a VIF of 1, implying a total absence of multicollinearity and ensuring the independence of these contributions to the model. These results support the validity of the model used, highlighting the relevance of the variables studied without indicating major concerns for multicollinearity.

**Table 6:** Collinearity test

**Relations**	**VIF**
ACCEP→INTU	3.351
BORE→INTU	2.944
EUS→INTU	3.765
INTU→INVERINFO	1.000
PIM→INTU	3.298
INVERINFO→RESPONUSE	1.000
INTERESTS→INTU	3.198
NEGEMO→INTU	1.209
POP→INTU	3.461
PRI→NTU	1.202
PUS→INTU	3.862
POSEMO→INTU	3.460

In Table 7, the analysis conducted using PLSpredict provides a detailed view of the model's predictive capability regarding three key latent variables: the intention for frequent use, the intention to verify information, and the responsible use of the ChatGPT. This analysis is crucial for understanding how the model performs in terms of accuracy and reliability when predicting these variables. Starting with INTU, we observe a *Q*^*2*^*predict*​ of 0.756, which is quite acceptable. This value indicates that the model has a robust predictive capacity for this variable, as it can explain a significant proportion of its variance. The accuracy of these predictions, as reflected in the RMSE (0.495) and MAE (0.377) values, confirms that the model achieves estimates quite close to the actual values, suggesting high reliability in this aspect. Similarly, for INVERINFO, *Q*^*2*^*predict*​ decreases to 0.5.

**Table 7:** PLSpredict RESULTS

**Construct**	Q^2^predict	RMSE	MAE
INTU	0.756	0.495	0.377
INVERINFO	0.5	0.509	0.384
RESPONUSE	0.469	0.531	0.402

Although this value still represents a moderate predictive capacity, it indicates that the model faces more difficulties when trying to predict this variable compared to the intention for frequent use. The RMSE and MAE values, which are slightly greater than those for the first variable, reinforce this observation, indicating an increase in the average error of the predictions. Finally, when analyzing RESPONSE, we find a *Q*^*2*^*predict*​ of 0.469. This value, while still indicating significant predictive capacity, is the lowest of the three, suggesting that the model finds the greatest difficulty in predicting behaviors related to the responsible use of the ChatGPT. Correspondingly, the RMSE and MAE values are the highest, indicating that the predictions for this variable are the least precise.

Table 8 includes the predictive capability test with cross-validation (CVPAT) using partial least squares structural equation modeling (PLS-SEM) versus the mean of indicators, providing a rigorous analysis of the model's predictive efficacy across different constructs. This approach compares the loss associated with PLS model predictions to that generated through a method based on the mean of indicators, thus offering a quantitative assessment of the model's predictive capability. For the INTU construct, we observe a PLS loss of 0.552, accompanied by a t value of 16.872 and a *p* value of 0.000. This result indicates the significantly robust predictive capability of the PLS model for this variable, with solid statistical evidence refuting the null hypothesis of ineffectiveness. Regarding INVERINFO, the PLS loss increases to 1.203, with a t value of 10.931 and a *p* value of 0.000. Despite the increase in predictive loss, the t and *p* values continue to demonstrate strong and statistically significant predictive capability, albeit with a larger margin of error compared to the intention for frequent use. The RESPONSE construct showed a PLS loss of 1.049, with a t value of 12.062 and a *p* value of 0.000. As in the case of the intention to verify information, this result indicates the notable predictive effectiveness of the model, though with slightly less accuracy than the first construct mentioned. Finally, when considering all the constructs together under the general category, the PLS loss is 0.956, with a t value of 15.835 and a p value of 0.000. This overall result reflects the strong general predictive capability of PLS-SEM, validating its utility in predicting a variety of constructs related to the use and perceptions of the ChatGPT. Overall, the results of the CVPAT test demonstrated that PLS-SEM has a significant predictive ability for each of the constructs examined, with robust statistical evidence supporting its efficacy. Although some constructs exhibit greater predictive loss than others, the consistency in statistical significance across all constructs underscores the validity of PLS-SEM as a reliable tool for prediction in this context.

**Table 8:** Predictive capability test with cross-validation (CVPAT) - PLS-SEM vs. mean of indicators

**Construct**	PLS loss	valor t	p valor
INTU	0.552	16.872	0.000
INVERINFO	1.203	10.931	0.000
RESPONUSE	1.049	12.062	0.000
General	0.956	15.835	0.000

### Hypothesis testing

**Fig. 2:**
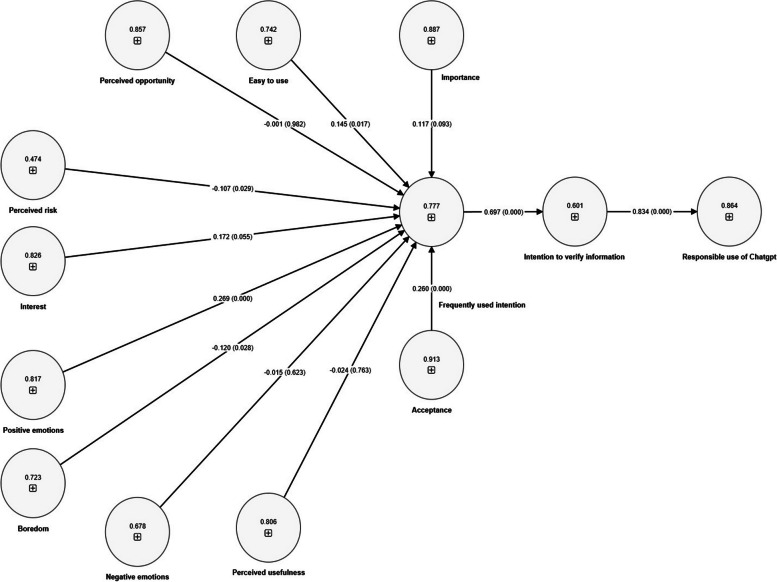
*P* values and standardized path coefficients of the relationships between the constructs of the research model

In the framework of this research, a series of hypotheses were tested via path regression analysis. The main objective was to analyze the relationships between several latent attitudinal variables of university students to understand their interaction and possible influence on the intention to frequently use ChatGPTs and responsible use. Table 9 and Fig. 2 detail the results obtained in this study, expressed in terms of parameter estimates (SE), standard errors, t values, and *p* values as well as the standardized path coefficients representing the relationships between the variables.

**Table 9:** Research hypothesis testing

**Path**	**Parameter estimations**	**SE**	**t values**	***p* value**	**Coefficients**	**Decision**
PIM→INTU	0.117	0.056	2.080	0.038*	0.193*	Supported
EUS→INTU	0.145	0.055	2.622	0.009**	0.204**	Supported
POP→INTU	-0.001	0.053	0.024	0.980	-0.002	Not Supported
PRI→INTU	-0.107	0.047	2.264	0.024**	-0.104**	Supported
INTERES→INTU	0.172	0.063	2.711	0.007**	0.238**	Supported
PUS→INTU	-0.024	0.062	0.388	0.698	-0.033	Not Supported
BORE→INTU	-0.120	0.052	2.302	0.022**	-0.145**	Supported
POSEMO→INTU	0.269	0.048	5.649	0.000***	0.418***	Supported
NEGEMO→INTU	-0.015	0.037	0.404	0.687	-0.017	Not Supported
ACCEP→INTU	0.260	0.047	5.515	0.000***	0.441***	Supported
INTU→INVERINFO	0.697	0.036	19.141	0.000***	0.509***	Supported
INVERINFO→RESPONUSE	0.834	0.030	28.006	0.000***	0.806***	Supported

Path Path coefficient, SE Standard error; ***p < 0.001; **p <0.01; *p <0.05

Nine of the twelve proposed hypotheses were confirmed. The path coefficients of the nine hypotheses tested ranged from -0.107 to 0.834; likewise, the *p* values of each of the hypotheses were statistically significant, confirming their influence on each of the proposed hypotheses. The hypothesis paths of POP, PUS, and NEGEMO are not statistically significant; therefore, H3, H6, and H9 are rejected.

## Discussion

This study investigated the underlying fundamentals of university students' attitudes toward ChatGPT. To this end, an SEM with acceptable validity and reliability indicators was developed. The results are framed within relevant theories, such as UTAUT2, the PATT-SQ-SE model, and Mitcham's philosophical framework of technology, and the practical implications for higher education institutions are debated, highlighting the need to improve curricula to leverage the benefits of AI and contribute to literacy in this field. The main findings are then contrasted with those of previous studies.

First, in relation to the cognitive component, the study showed that PIM positively influences INTU [62], suggesting that students' perception of the importance of the use of ChatGPT as a learning tool is a relevant factor in determining their intention to use it frequently. Thus, it can be inferred that students who consider ChatGPT a valuable tool for their learning process are more likely to use it more frequently than are those who do not perceive its importance. EUS was also found to have a significant influence on the INTU of the ChatGPT system, indicating that, in general, users find this generative artificial intelligence platform easy to use and are, therefore, more likely to use it frequently. This is a critical factor to consider for the success of any chat system, as it is important for users to easily navigate the system and quickly and efficiently find what they need. Therefore, chat system designers must pay particular attention to ease of use to ensure that users have a satisfactory experience and want to continue using the system in the future. The above hypotheses were also tested in studies with technology users in a variety of contexts [30, 31, 40, 42, 63–65].

Privacy issues are of concern to students, as RISK has been found to have a negative impact on INTU. Therefore, developers of generative artificial intelligence systems must consider users' concerns and implement solutions that ensure the privacy of their data. It is critical that users feel secure when using an application and trust that their personal data will not be shared or used inappropriately. These results coincide with those of [11, 63, 68–70]. However, the study also showed that POP does not positively influence INTU, suggesting that users may be motivated by various reasons for using a product or service. The perception of an opportunity may simply be curiosity or a momentary need, whereas habitual use intention may be motivated by a more constant need or personal preference.

Regarding the constructs of the affective component of attitude, the study corroborates the significant influence of INTEREST and INTU, suggesting that students who show a greater interest in using ChatGPT in their academic activities are more likely to use it more frequently [60]. In addition, BORE has a significant influence on students' INTU when using ChatGPT, indicating that students who are less bored tend to use some technology more frequently. This hypothesis was also tested by [27]. [72] reported that participants endorsed high-frequency positive statements about the beneficial applications of AI in the community. Moreover, few participants expressed negative emotions toward the AI in negative items. The present study showed that POSEMO positively influences the INTU of student users of ChatGPT, while the hypothesis that NEGEMO influences the intention to use frequently does not have a significant influence, implying that positive emotions are more important determinants of the intention to use a generative artificial intelligence tool frequently. It is of utmost importance to highlight that the user experience on a technological platform is based not only on efficiency and application but also on the emotional satisfaction it provides. Therefore, it is essential that HEIs consider the importance of positive emotions in the user experience in the implementation process of teaching and learning. These results coincide with those of Schepman and Rodway, in which most participants supported the POSEMO items compared to the NEGEMO items. This shows that university students positively perceive the immersion of AI tools in higher education (such as the ChatGPT), valuing more of their positive aspects. In contrast to the findings of previous studies [30, 31, 42, 65], in this study, PUS did not influence INTU. A probable cause may be the unit of analysis and the context of this study.

Third, regarding the behavioral component, the study confirmed that the ACCEP of the ChatGPT influences learners' INTU, indicating that users who accept and feel comfortable with the ChatGPT platform are more likely to use it more frequently. This result highlights the relevance of user experience in the implementation of educational technologies and agrees with those of [64, 73, 74]. Furthermore, a significant influence was demonstrated between INTU and INVERINFO obtained from ChatGPT by students, implying that students who intend to use ChatGPT frequently also intend to verify the information they obtain from this tool. This shows that students feel aware that it is important to verify the information and take steps to ensure that the information provided by this system is accurate and reliable. This hypothesis has been tested in previous studies [75, 77].

Similarly, the study demonstrated the significant influence of ChatGPTs’ INVERINFO and RESPONUSE on students' academic activities, implying that students who have a greater intention to verify information and responsible use of ChatGPTs tend to perform better in their academic activities. This finding suggested that using generative artificial intelligence tools, such as ChatGPT, can be beneficial to students in their learning process. Likewise, the study highlights the relevance of fostering digital literacy so that students can make adequate and effective use of these technological tools in their academic and professional environments. The latter is located within the recommendations of [82], which explains that educational actors should make ethical and responsible use of the system.

The findings of this study are significant for the field of higher education because they provide valuable insights into the factors influencing students' intentions to use ChatGPT as a learning tool. Additionally, the study underscores the importance of cognitive and affective components and their impact on the behavioral component of students' use of ChatGPT in their academic activities.

These results are decisive and serve as a reference framework for the design and implementation of educational technologies, especially in the context of higher education institutions (HEIs). Moreover, by considering these factors, the effectiveness and efficiency of teaching and learning procedures can be optimized, offering students a more engaging and rewarding learning experience.

Specifically, HEIs can leverage these findings to develop strategies that promote the responsible and ethical use of ChatGPT, addressing concerns about data privacy and information verification. Furthermore, they can encourage positive emotions and student interest in using this tool while mitigating boredom and negative emotions. Finally, HEIs can integrate ChatGPTs into their institutional platforms, such as virtual classrooms and tutoring systems, taking into account the perceived importance and ease of use of this technology.

## Conclusions

In conclusion, as the application of generative AI tools continues to increase in higher education, it is essential to explore the constructs that affect students' attitudes when engaging in university activities. The present study contributes to the unified theory of attitude toward the ChatGPT-UTAC with Mitcham's philosophical framework [60], reinforcing the predictive capability of the PATT-SQ-SE model [48] and UTAUT2 [28].

The primary objective was to analyze university students’ attitudes toward the ChatGPT. Therefore, 13 constructs from the proposed hybrid model were examined, formulating 12 hypotheses from these constructs, nine of which were accepted. Regarding the analysis of the effect of the intention to use frequently, it has been established that the intention to use frequently has a significant impact on the intention to verify information and affects the responsible use of the ChatGPT. Therefore, a greater frequency of ChatGPT use constitutes a determining factor for the intention to verify the information provided by this tool. These findings suggested that university students who regularly interact with ChatGPTs tend to develop a critical sense of the information obtained, possibly due to increasing familiarity with their capabilities and limitations. Similarly, the direct effect between frequent use intention and responsible use of ChatGPT indicates that students are more aware of the importance of using this technology ethically and responsibly.

On the other hand, the study revealed that students' perceptions of the importance of ChatGPT and its ease of use are decisive factors in determining their intention to use it frequently. Concerns about privacy also negatively influenced students' intentions to use ChatGPT, highlighting the importance of implementing solutions that ensure the privacy of user data. Additionally, the study suggested that positive emotions, such as interest and positive affect, play a significant role in students' intentions to use ChatGPT. The study also emphasized the importance of promoting digital literacy among students to enable them to use generative artificial intelligence tools such as ChatGPT effectively in their academic and professional environments. Overall, this study provides a comprehensive framework on how educational institutions can implement generative AI tools in the teaching-learning process to enhance students' learning experiences. Finally, a primary novelty of this study lies in the adaptation and validation of preexisting theoretical models, such as the UTAUT2 and the PATT-SQ-SE models, in the specific context of ChatGPT adoption in higher education. Moreover, the incorporation of constructs such as responsible use and the intention to verify information broadens the understanding of how frequency of use influences a more critical relationship with AI. These findings provide a solid foundation for future research on the impact of ChatGPT in higher education and how institutions can effectively leverage this technology.

## Practical and theoretical implications

The practical implications of this study are as follows: HEIs should consider acceptance to improve their educational curricula and contribute to AI literacy. In addition, the ease of use and importance of the ChatGPT can support its integration into institutional platforms such as virtual classrooms and the implementation of tutoring systems based on AI. The perceived risk should serve for academic authorities of HEIs to manage systems that support the management of personal data to reduce the risks of using AI tools.

On the other hand, this study contributes to the understanding of student attitudes toward these technologies (such as ChatGPT), which is crucial for HEIs to design and ensure educational experiences that are both engaging and effective. By recognizing the factors influencing students' acceptance and use of ChatGPTs, HEIs can adopt more personalized approaches that align artificial intelligence tools with student needs and expectations.

Furthermore, the practical value of this study allows HEIs not only to integrate ChatGPT and other AI tools more strategically into their curricula but also to design specific interventions to improve AI literacy in the student community. For example, workshops, seminars, and elective courses on AI could be incorporated to equip students with the critical skills necessary to effectively evaluate and utilize these technologies.

Regarding theoretical implications, the current study contributes to the unified theory of acceptance toward ChatGPT (UTAC), which comprises 13 constructs (significance, opportunity, ease of use, perceived risk, intention to use frequently, interest, boredom, acceptance, perceived utility, positive and negative emotions, intention to verify information, and responsible use of ChatGPT) that are grounded in the attitude components of Mitcham's philosophical framework of technology [60], the PATT-SQ-SE model [48], and the UTAUT2 [28].

This theory contributes to the field of educational technology and serves as a reference for future research aimed at evaluating university students' attitudes toward a specific educational technology. Furthermore, the inclusion of constructs such as the responsible use of the ChatGPT and the intention to verify information reflect the growing awareness of the importance of critical digital literacy among university students, where ethical and responsible use is prioritized to effectively leverage AI opportunities. By analyzing how these elements affect the acceptance and use of technological tools, this study broadens the understanding of the cognitive and affective processes underlying technological adoption.

## Limitations and future studies

The main limitations of the study that serve as references for future research are as follows. First, an incidental convenience sample was used; therefore, it is likely that the findings do not apply to the general population of higher education students [89]. On the other hand, the participants belonged to universities located in the main urban areas of the country where internet access and technological resources are optimal in comparison to HEIs located in rural areas where the results probably cannot be applied. In addition, since there were no scales available to measure attitudes toward the ChatGPT, the items had to be adapted from previous studies, and although pretests were conducted to determine validity and reliability, the responses in some constructs of the scale are not free of biases.

In future research, this study could serve as a reference for creating scales to measure attitudes toward specific generative AI tools such as the ChatGPT. In addition, future studies can integrate new constructs such as effort-performance expectancy, social influence, hedonic motivation, and price value to more comprehensively explore the attitudes of all stakeholders involved in higher education (teachers, students, and managers) to efficiently take advantage of the benefits of AI and decrease its negative impacts.

### Supplementary Information

Supplementary Material 1. 

Supplementary Material 2. 

## Data Availability

The datasets used and/or analyzed during the current study are available from the corresponding author upon reasonable request.

## Abbreviations

AI: Artificial intelligence
HEIs: Higher education institutions
UTAUT2: The unified theory of acceptance and use of technology
PATT-SQ-SE: Pupil Attitudes Toward Technology Model
GAAIS: General Attitudes Toward Artificial Intelligence Scale
AVE: Average variance extracted
CR: Composite reliability
HTMT: Heterotrait–monotrait relationship index test
