# Supplementary material for: Analysis of college students' attitudes toward the use of ChatGPT in their academic activities: effect of intent to use, verification of information and responsible use
Source: BMC Psychol. 2024 May 8;12:255. doi: 10.1186/s40359-024-01764-z (PMC11077796; doi:10.1186/s40359-024-01764-z)
Supplement: Supplementary file 1 — Supplementary Material 1. [file 40359_2024_1764_MOESM1_ESM.docx]

**Appendix-1**. Data collection instrument

| **Construct** |  | **Items** |
| --- | --- | --- |
| Importance | PIM1  PIM2  PIM3  PIM4 | Using ChatGPT in my academic activities is important because it enhances my learning experience.  ChatGPT improves my productivity and efficiency in the execution of academic activities.  The use of ChatGPT in my academic activities contributes to the efficiency and effectiveness of my learning process.  ChatGPT is a tool that improves my ability to develop academic projects and activities. |
| Ease of use | EUS1 EUS2  EUS3  EUS4 | The ChatGPT interface is user-friendly and easy to use.  I find it easy to adapt ChatGPT responses to what I need to do my academic work and/or projects.  I have difficulty selecting relevant information from the answers generated by ChatGPT.  I find it easy to formulate prompts in ChatGPT to get answers according to my needs. |
| Perceived opportunity | POP1  POP2  POP3 | Using ChatGPT in my academic activities allows me to develop projects and tasks more efficiently and effectively.  Using ChatGPT in my academic activities develops my autonomous and self-directed learning skills.  Using ChatGPT in my academic activities enables me to explore different perspectives and approaches to my course content. |
| Perceived risk | PRI1  PRI2  PRI3 | Frequent use of ChatGPT, diminishes my abilities to think critically and solve problems independently.  I am aware that not all answers provided by ChatGPT are correct and may include biases.  Frequent use of ChatGPT poses a threat to the privacy and security of my personal data. |
| Perceived interest | INTERES1  INTERES2  INTERES3  INTERES4 | I consider it pertinent to use ChatGPT as a complementary tool in my academic activities.  I am attracted to the possibility of using ChatGPT to improve my productivity and efficiency in the development of projects and/or assignments.  I find the use of ChatGPT in my academic activities exciting and enhances my learning process.  I am enthusiastic about using ChatGPT to find solutions and answers to my academic questions and concerns. |
| Boredom | BORE1 BORE2  BORE3 | I find it boring to use ChatGPT in my academic activities.  Frequent use of ChatGPT gives me a feeling of boredom and disinterest in learning.  Using ChatGPT gives me a feeling of monotony and boredom in the way I get information and answers. |
| Perceived usefulness | PUS1  PUS2  PUS3 | ChatGPT is a valuable tool to improve my academic performance and achieve more satisfactory results in my studies.  ChatGPT offers me practical and useful solutions to face academic and/or personal challenges and tasks.  ChatGPT is a useful tool to understand and comprehend complex topics in my subjects. |
| Acceptance | ACEPP1 ACEPP ACEPP3  ACEPP4 | I am willing to use ChatGPT as a tool to improve my academic performance.  I am willing to use ChatGPT as part of my learning process.  I consider it appropriate and beneficial to use ChatGPT as a tool to support my academic and/or personal activities.  I am open to using ChatGPT as part of my learning process at the university. |
| Intended use | INTU1 INTU2  INTU3 | I will use ChatGPT frequently in my academic activities for a long period of time.  I will frequently and continuously use ChatGPT as a virtual expert to guide me in my academic activities.  I am willing to use ChatGPT frequently in my academic activities. |
| Intention to verify information | INVERINFO1  INVERINFO2   INVERINFO3 | I am willing to verify the information obtained from ChatGPT through additional sources before considering it as completely accurate or true.  I am willing to corroborate the information provided by ChatGPT through comparison with academic sources and experts in the relevant field.  It is not necessary to verify the veracity of the information provided by ChatGPT because it always provides valid and reliable information. |
| Positive emotions | POSEMO1 POSEMO2 POSEMO3 | Artificial intelligence systems like ChatGPT can help students feel happier.  I am impressed by what I can do using ChatGPT in my academic activities.  I believe that ChatGPT can have positive effects on students' well-being. |
| Negative emotions | NEGEMO1  NEGEMO2  NEGEMO3 | I dislike the idea that technology such as ChatGPT replaces certain human skills such as inferring, information seeking, analyzing, writing, etc.  I am concerned that frequent use of ChatGPT will limit my ability to think and solve problems independently.  I am concerned that excessive use of ChatGPT will diminish my interest in researching and reading diverse sources of information. |
| Responsible use of ChatGPT | RESPONUSE1  RESPONUSE2   RESPONUSE3   RESPONUSE4  RESPONUSE5 | When using ChatGPT I carefully check your responses to ensure that they are correct, complete, and free of bias.  I use ChatGPT responsibly by not presenting technology-generated responses as if they were my own work product, without proper attribution.  I use ChatGPT responsibly and ethically, ensuring that the answers obtained are a tool to support my learning and not a replacement for my own intellectual effort.  I use ChatGPT responsibly and ethically, avoiding the generation of misleading, false and/or biased content.  I strive to understand the limitations of ChatGPT and its potential to generate incorrect or biased responses, which motivates me to use it with caution and discernment. |
