# Supplementary material for: Analysis of college students' attitudes toward the use of ChatGPT in their academic activities: effect of intent to use, verification of information and responsible use
Source: BMC Psychol. 2024 May 8;12:255. doi: 10.1186/s40359-024-01764-z (PMC11077796; doi:10.1186/s40359-024-01764-z)
Supplement: Supplementary file 2 — Supplementary Material 2. [file 40359_2024_1764_MOESM2_ESM.docx]

**Supplementary Material**

The following table shows the standardized internal weights of the collection instrument

| **Ítems** | **External loads (standardized)** |
| --- | --- |
| P1<-PIM | 0.941 |
| P10<-POP | 0.883 |
| P11<-POP | 0.939 |
| P12<-PRI | 0.691 |
| P13<-PRI | 0.632 |
| P14<-PRI | 0.739 |
| P15<-INTEREST | 0.829 |
| P16<-INTEREST | 0.940 |
| P17<-INTEREST | 0.940 |
| P18<-INTEREST | 0.922 |
| P19<-BORE | 0.810 |
| P2<-PIM | 0.951 |
| P20<-BORE | 0.826 |
| P21<-BORE | 0.912 |
| P22<-PUS | 0.884 |
| P23<-PUS | 0.912 |
| P24<-PUS | 0.897 |
| P25<-ACCEP | 0.954 |
| P26<-ACCEP | 0.966 |
| P27<-ACCEP | 0.956 |
| P28<-ACCEP | 0.946 |
| P29<-INTU | 0.893 |
| P3<-PIM | 0.946 |
| P30<-INTU | 0.882 |
| P31<-INTU | 0.870 |
| P32<-INVERINFO | 0.963 |
| P33<-INVERINFO | 0.936 |
| P34<-INVERINFO | 0.046 |
| P35<-POSEMO | 0.927 |
| P36<-POSEMO | 0.900 |
| P37<-POSEMO | 0.884 |
| P38<-NEGEMO | 0.774 |
| P39<-NEGEMO | 0.820 |
| P4<-PIM | 0.929 |
| P40<-NEGEMO | 0.873 |
| P41<-RESPONUSE | 0.920 |
| P42<-RESPONUSE | 0.926 |
| P43<-RESPONUSE | 0.952 |
| P44<-RESPONUSE | 0.946 |
| P45<-RESPONUSE | 0.903 |
| P5<-EUS | 0.882 |
| P6<-EUS | 0.908 |
| P7<-EUS | 0.785 |
| P8<-EUS | 0.866 |
| P9<-POP | 0.953 |
